# Analysis of Gaseous and Particle-Associated PAH and Nitroarenes in Ambient Air

**DOI:** 10.6028/jres.093.044

**Published:** 1988-06-01

**Authors:** Janet Arey, Barbara Zielinska, Roger Atkinson, Arthur M. Winer

**Affiliations:** Statewide Air Pollution Research Center University of California Riverside, CA 92521

The organic species present in ambient air vary considerably in volatility and concentration and are distributed between the gaseous and particulate phases. This distribution depends on, among other factors, ambient temperature and the composition of the particulate phase. The major sources of anthropogenic chemicals emitted into the atmosphere are combustion and volatilization, and the more volatile species are often present in high concentrations. This is demonstrated in figure 1 for a series of aromatic hydrocarbons. Literature values for ambient benzene and toluene concentrations [1] and the ambient concentration data we obtained for polycyclic aromatic hydrocarbons (PAH) at Glendora, CA, in August 1986 are shown plotted against their room temperature vapor pressures [2,3]. Thus, to measure ambient concentrations of the complete range of PAH and nitroarenes (which are generally at least an order of magnitude less abundant than the parent PAH), complementary sampling and analysis techniques are required.

Indicated in table 1 are the sampling technique(s) which provided quantitative concentration values-for the listed PAH during 12-h sampling periods. The sampling techniques employed were: sampling at 1 L min^−1^ onto cartridges containing 0.1 g of Tenax GC solid adsorbent (low-flow Tenax), sampling at 10 L min^−1^ onto 0.6 g of Tenax (high-flow Tenax), sampling at 30 SCFM onto four polyurethane foam (PUF) plugs located in series downstream from Teflon-impregnated glass fiber (TIGF) filters in modified high-volume samplers, and standard high-volume sampling at 40 SCFM onto TIGF filters. Crucial to the quantification procedures was the utilization of a complete range of deuterated internal standards, from naphthalene-d_8_ to dibenz(a,h)anthracene-d_14_, added to the appropriate samples prior to the extraction step.

Generally the abundant volatile ambient nitroarenes, which include 1- and 2-nitronaphthalene and 3-nitrobiphenyl, are efficiently sampled utilizing PUF plugs [4]. To understand the sources of the nitroarenes observed in ambient air, isomer-specific compound identification is required [5,6]. The identification in an ambient particulate extract of the most abundant particle-associated nitroarene species as 2-nitrofluoranthene is illustrated in figure 2. Shown in this figure are the gas chromatographic/mass spectrometric (GC/MS) multiple ion detection (MID) traces for the molecular ion (*m*/*z* 247) of the isomeric nitrofluoranthenes (NF), nitropyrenes (NP) and nitroacephenanthrylenes (NAce) and for the molecular ion (*m*/*z* 256) of the corresponding perdeuterated species added as internal standards. The GC/MS identification of 2-NF and additional NF, NP and NAce isomers in ambient samples has been achieved through the use of deuterated internal standards in conjunction with the consistent chromatographic resolution and high sensitivity achievable through sample clean-up by high performance liquid chromatographic separations [7,8].

## Figures and Tables

**Figure 1:**
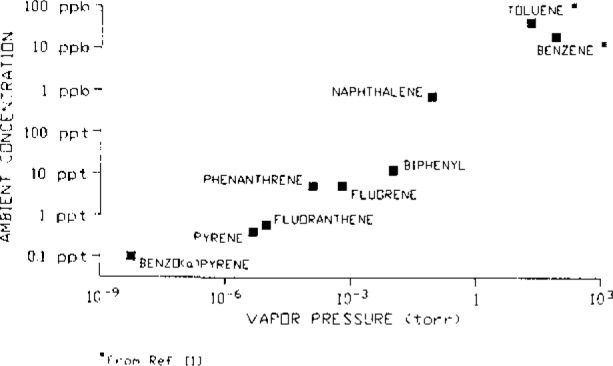
Plot of typical ambient concentrations of a series of aromatic hydrocarbons as a function of their room temperature vapor pressures.

**Figure 2:**
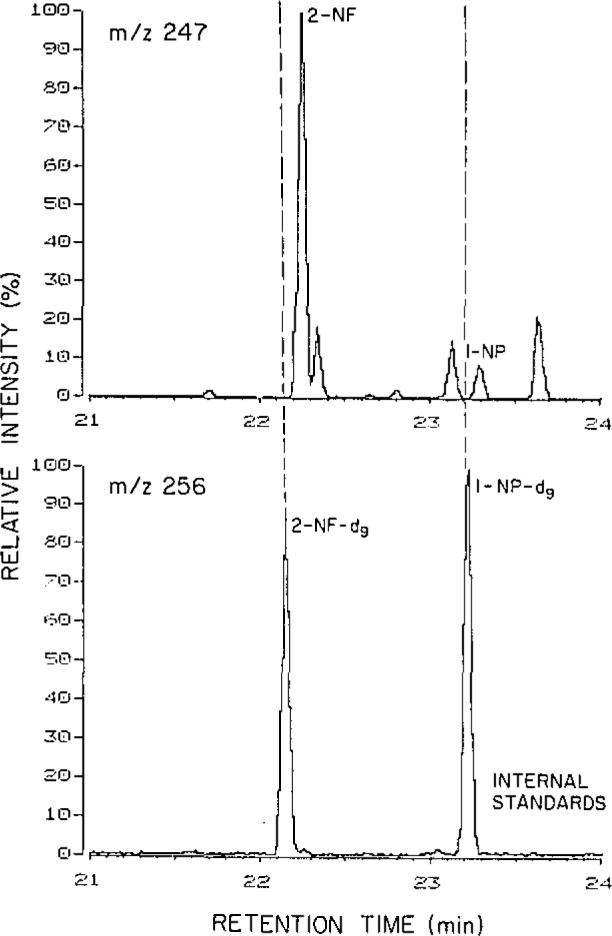
GC/MS MID traces of an ambient particulate sample extract showing that the most abundant NF, NP or NAce isomer is 2-nitrofluoranthene.

**Table 1:** PAH quantitatively measured and the sampling techniques employed at Glendora, CA, in August 1986

m.w.	PAH	Low-flow Tenax	High-flow Tenax	PUF plugs	Filter	Comment
128	Naphthalene	X				Ave. 2% on back-up Tenax
142	1-Methylnaphthalene	X	X			Low- and high-flow Tenax agree
142	2-Methylnaphthalene	X	X			Low- and high-flow Tenax agree
154	Biphenyl		X			Too low to quantify on low-flow Tenax
154	Acenaphthene		X			Too low to quantify on low-flow Tenax
166	Fluorene		X			ΣPUFs ~25% high-flow value
178	Phenanthrene		X	X		<10% on 4th PUF
178	Anthracene			X		Too low to quantify on high-flow Tenax
184	Dibenzothiophene			X		
202	Fluoranthene			X		Partially gas-phase Partially “blown-off” filter <10% on filter
202	Pyrene			X		Partially gas-phase Partially “blown-off” filter <10% on filter
228	Chrysene/Triphenylene			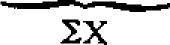		20–50% present on PUFs
Higher Molecular Weight PAH					X	
